# TARGETED PRINCIPLE COMPONENT ANALYSIS: A NEW MOTION ARTIFACT CORRECTION APPROACH FOR NEAR-INFRARED SPECTROSCOPY

**DOI:** 10.1142/S1793545813500661

**Published:** 2013-12-02

**Authors:** MERYEM A. YÜCEL, JULIETTE SELB, ROBERT J. COOPER, DAVID A. BOAS

**Affiliations:** * Athinoula A. Martinos Center for Biomedical Imaging, Department of Radiology, Massachusetts General Hospital, Harvard Medical School, Charlestown, MA, USA; † Department of Medical Physics and Bioengineering, University College London, London, UK

**Keywords:** Wavelet, spline, collodion-fixed fiber

## Abstract

As near-infrared spectroscopy (NIRS) broadens its application area to different age and disease groups, motion artifacts in the NIRS signal due to subject movement is becoming an important challenge. Motion artifacts generally produce signal fluctuations that are larger than physiological NIRS signals, thus it is crucial to correct for them before obtaining an estimate of stimulus evoked hemodynamic responses. There are various methods for correction such as principle component analysis (PCA), wavelet-based filtering and spline interpolation. Here, we introduce a new approach to motion artifact correction, targeted principle component analysis (tPCA), which incorporates a PCA filter only on the segments of data identified as motion artifacts. It is expected that this will overcome the issues of filtering desired signals that plagues standard PCA filtering of entire data sets. We compared the new approach with the most effective motion artifact correction algorithms on a set of data acquired simultaneously with a collodion-fixed probe (low motion artifact content) and a standard Velcro probe (high motion artifact content). Our results show that tPCA gives statistically better results in recovering hemodynamic response function (HRF) as compared to wavelet-based filtering and spline interpolation for the Velcro probe. It results in a significant reduction in mean-squared error (MSE) and significant enhancement in Pearson’s correlation coefficient to the true HRF. The collodion-fixed fiber probe with no motion correction performed better than the Velcro probe corrected for motion artifacts in terms of MSE and Pearson’s correlation coefficient. Thus, if the experimental study permits, the use of a collodion-fixed fiber probe may be desirable. If the use of a collodion-fixed probe is not feasible, then we suggest the use of tPCA in the processing of motion artifact contaminated data.

## 1. Introduction

Near-Infrared Spectroscopy (NIRS) can measure oxygenation and blood volume changes in tissue by detecting the optical property changes within.^1^ The technique is finding widespread application for studying cerebral physiology and brain activation due to its noninvasiveness, portability and long-term recording ability. NIRS has been used both in the clinic for monitoring purposes^2,3^ and in research on brain and muscle tissue.^4,5^ The technique has also found use in the cognitive neurosciences^6^ and in the study of various neurological disorders such as epilepsy, depression and Alzheimer’s disease.^7–10^

As the applications broaden to different age and disease groups, motion artifacts in the NIRS signal due to subject movements become an important challenge. There are mainly two approaches to overcome this problem: improve the scalp–optode coupling using various probe designs and/or remove or correct motion artifacts in the signal using postprocessing methods. Examples for the first category are brush optodes,^11^ mechanical mounting structures,^12^ modified cycle helmets, thermoplastics moulded to the contours of subject’s head, spring-loaded fibers^13^ and collodion-fixed fiber-based probes.^14^

The second approach involves various post-processing motion artifact correction methods. These methods either require an external measurement of the movements that is then incorporated into an adaptive filtering algorithm^15^ or they use spatial and/or temporal features of the NIRS signal itself, as is the case for wavelet-based filtering,^16^ principle component analysis (PCA),^17^ spline interpolation^18^ or Kalman filtering.^19^ Comparison of the methods has shown that the most effective methods for motion artifact correction are wavelet-based filtering and spline interpolation.^20,21^

In this study, we introduce a new approach for motion artifact correction: targeted principle component analysis (tPCA) which is a modified version of regular PCA. The method applies PCA iteratively only on the pre-determined epochs of data that contain motion artifacts. We compare the new method with the most effective methods previously identified in literature, namely wavelet-based filtering and spline interpolation. We also compare the motion artifact corrected NIRS signal obtained with a standard Velcro NIRS probe vs a more stable collodion-fixed NIRS probe, to assess the effectiveness of post-processing to correct motion artifacts vs the more time consuming approach of creating a NIRS probe less susceptible to motion artifacts.

## 2. Methods

### 2.1. NIRS data

The experimental protocol and the data used in this work have been previously reported.^14^ Five healthy adult subjects were recruited for this study (1 female, 4 male; 23–52 years old). The study included collection of NIRS data from collodion-fixed optical fibers (left motor region) and Velcro-based probe (right motor region) during the performance of several commonly encountered motion artifacts. The collodion-fixed fiber tip consists of a glass prism, a mirrored surface and a prism-housing. Each optode was coupled to the head with the help of a clinical adhesive (Collodion, Mavidon, FL) so as to symmetrically match the standard probe. See Ref. 14 for more details about the probes and geometry. The study was approved by Massachusetts General Hospital and each subject gave written consent.

**Fig. 1:**
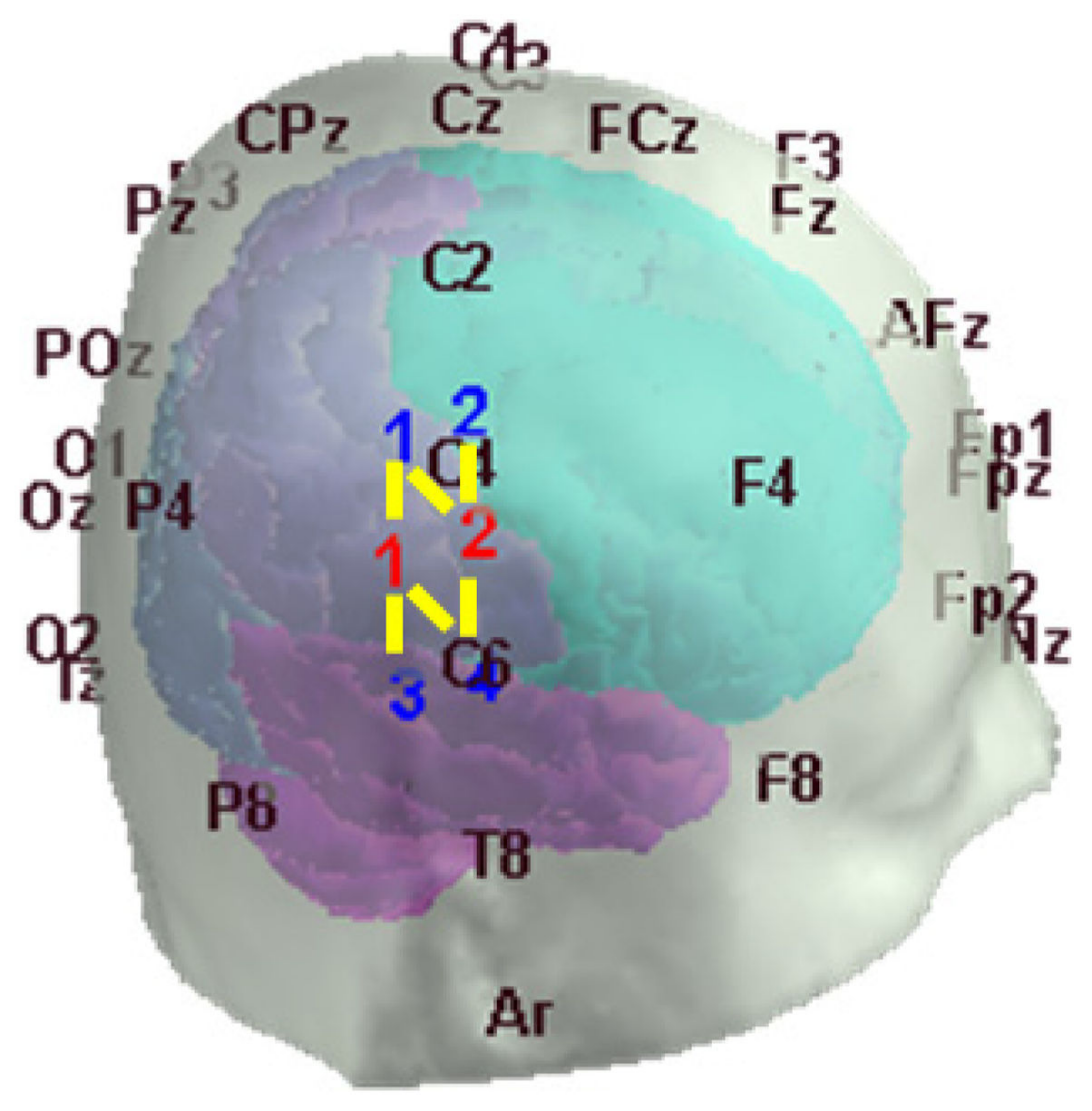
Optical probe. Symmetrical source detector localization on both hemispheres. The red and blue numbers indicate the position of sources and detectors, respectively. The yellow lines indicate a source–detector pair.

Data were obtained using a TechEn CW6 system (Medford, MA, USA). Each probe contained two sources and four detectors (See Fig. 1). Throughout the 6-min recording, subjects performed each of the following movements five times: reading aloud, nodding their head up and down, nodding sideways, twisting upper body right, twisting upper body left, shaking head rapidly from side to side and raising their eyebrows (randomized inter-trial interval between 5 and 10 s). The Psychophysics toolbox for MATLAB was used to control the timing of each motion trial.^22^

### 2.2. Motion correction methods

#### 2.2.1. Spline interpolation

The spline interpolation method models the period of motion artifacts in the data via cubic spline interpolation.^18^ The periods of motion artifacts are automatically determined on a channel-by-channel basis using hmrMotionArtifactByChannel function under the HOMER2 package.^23^ The interpolated motion artifact segment is first subtracted from the original segment. The resulting segments in the time series are then shifted by a value given by the mean value of the segment and the mean value of the preceding segment. We used a spline interpolation parameter of 0.99 as in a previous work by Scholkmann *et al.*^18^

#### 2.2.2. Wavelet filtering

The discrete wavelet analysis and filtering approach described by Molavi and Dumont^16^ was used in this study. The wavelet method first decomposes the time-course into the wavelet domain using the general discrete wavelet transformation. The model assumes that the wavelet coefficients have a Gaussian probability distribution, with physiological components centered around zero and motion artifacts appearing as outliers. The tuning parameter *α* determines the threshold for motion artifact determination. The parameter is set to 0.1 in this work as in Molavi and Dumont.^16^ The Wavelab 850 toolbox (www-stat.stanford.edu/~wavelab) for MATLAB was employed during the analysis.

#### 2.2.3. Targeted principle component analysis

Regular PCA applies an orthogonal transformation to convert a matrix of measurements, where the matrix is generally arranged as number of time points by number of channels, into a set of orthogonal vectors. It assumes that motion artifacts appear in multiple channels with a common temporal variation, and that motion artifacts compose the majority of the variation in the signal.^17^ A certain number of principle components, ranked in decreasing order of the percent variance of the data that they explain, are then projected out of the data.

As opposed to standard PCA, tPCA applies the same procedure only on the periods of data identified to have motion artifacts. If a motion artifact is identified automatically on any channel, then the epoch of data is included in the PCA for all channels. The epochs of motion are combined into a single data matrix (time points by channels). The principle components are ranked in decreasing order of percent variance explained and then a certain number of components are projected out of the data. We project out *N*_tPCA_ components to remove up to 97% of the variance in the data. These epochs of corrected motion are then stitched back into the original data time series by shifting the mean value of adjacent epochs of motion and motion free data to align the adjoining time points. This shifting procedure is identical to the procedure used in the spline method of motion correction as described in detail in Scholkmann *et al*.^18^ We repeat this procedure two times, re-identifying any residual motion artifacts, to further filter any that had not been fully corrected. The three iterations are chosen as further iterations do not provide further improvement in the results.

#### 2.2.4. Processing stream

A synthetic hemodynamic response function (HRF) was generated in the raw NIRS data by introducing a signal change of 0.9% from baseline for the 690-nm channels and 2% for 830-nm channels. The HRF was a gamma function which peaks around 6 s and lasts for 16 s with a 10-*μ*M increase in HbO and 4-*μ*M decrease in HbR. A pathlength correction factor of 6 was applied.^24^ This HRF was added to the data channels collected for each subject with an inter-stimulus interval that ranged from 5 to 10 s providing 17 to 19 stimulus trials per 6-min run of data. A total of 25 different random stimulus onsets were used for each subject to further randomize the timing of stimulus onset with respect to the motion artifacts. Following the addition of the HRF, the raw NIRS data were first converted into changes in optical density. Then motion artifacts were identified automatically using the hmrMotionArtifact-ByChannel function in Homer2. This function identifies motion artifacts on a channel by channel basis using a set of pre-determined thresholds for changes in absolute signal amplitude (AMPthresh) or changes relative to the standard deviation of the data (STDthresh) within a given period of time (tMotion), and marks a time range (tMask) around the motion artifact as a motion artifact mask. The values used in this study were AMPthresh = 5, STDthresh = 20, tMotion = 0.5 s and tMask = 1 s. Following the determination of epochs of motion artifacts, the motion artifact correction method was applied. The data were then band-pass filtered with a third-order Butterworth filter between 0.01 and 0.5 Hz to remove low-frequency drifts and high-frequency noise. Finally, a simple block average was performed to estimate the mean HRF.

#### 2.2.5. Metrics

We used two metrics to compare the efficacy of each method. We computed the mean-squared error (MSE) and Pearson’s correlation coefficient (*R*^2^) between the HRF obtained after applying each motion correction method and the true HRF.

## 3. Results

**Fig. 2:**
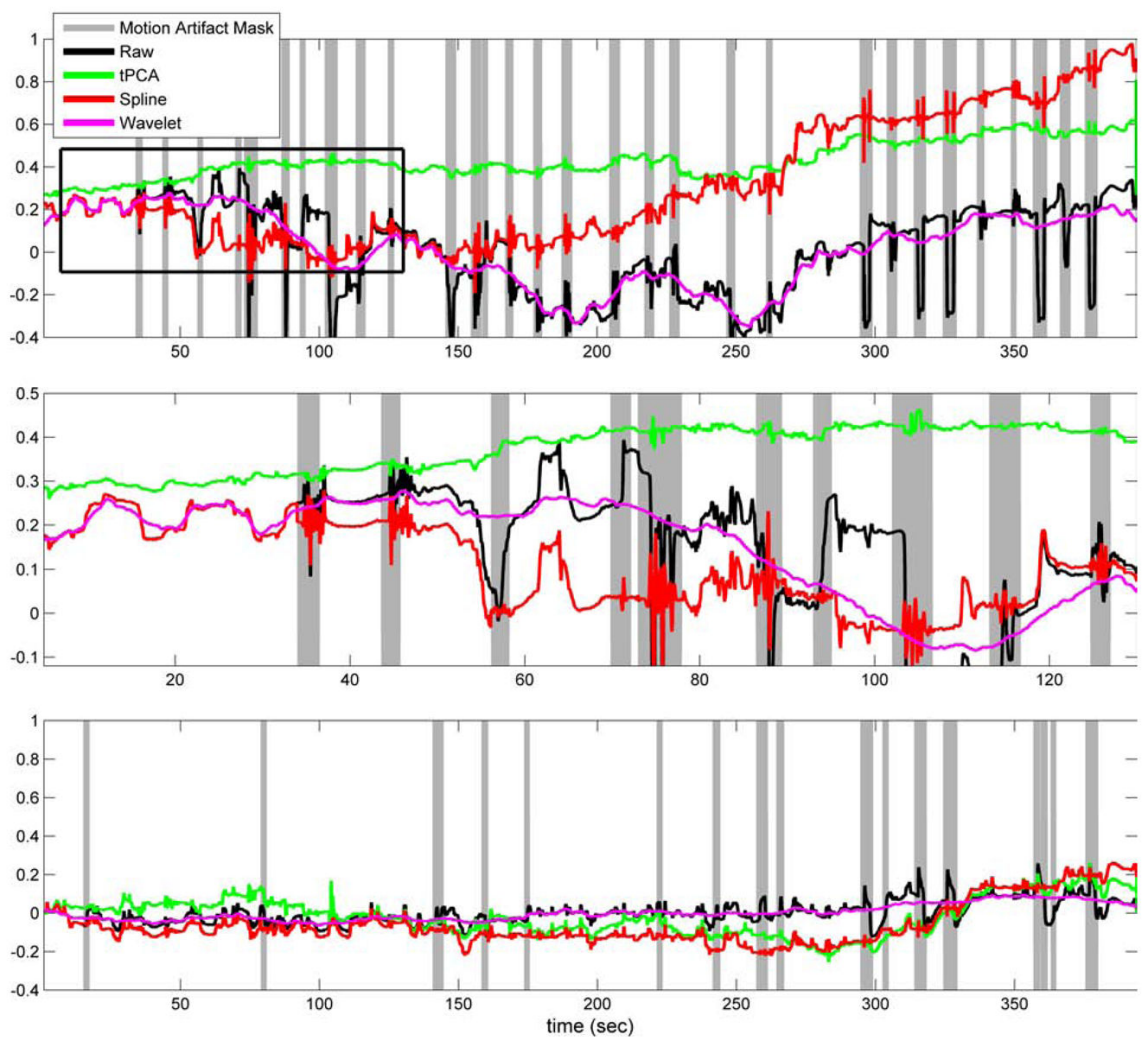
Optical density change at 690 nm for a channel from the velcro-based probe (top panel) and from the collodion-fixed probe (bottom panel) for one subject with no synthetic HRF added (black: unprocessed data; green: tPCA corrected data; red: spline interpolation corrected data; pink: wavelet-based filtered data). Inset box is zoomed in the middle panel. The gray-shaded areas show the motion artifact mask identified in the corresponding channel only.

Figure 2 shows an example of the changes in optical density before (raw) and after the application of motion artifact correction methods (tPCA, spline and wavelet-based filtering) for one subject. The channel shown in Fig. 2 is from the Velcro-based probe. The signal obtained is highly contaminated by motion artifacts as opposed to channels of data acquired with the collodion-fixed fiber probe where each optode is tightly glued on the head of the subject. Motion artifacts induced by subject movement are clearly seen and automatically identified in the raw data. This time series exemplifies the impact of the different motion artifact correction methods on the optical density changes. Note that the spline result follows the raw data until ~35 s when the first motion artifact is identified for this channel. Beyond ~35 s, we see divergence of spline from the raw data because of spline motion correction. The wavelet motion correction causes an earlier divergence from the raw data because of the different methods applied to remove wavelet coefficients affected by motion artifacts. There different methods have thus corrected the signal in the first few seconds of data acquisition which the wavelet method identified as motion. The same is true for the tPCA which instead of utilizing motion artifacts identified on this single channel, is utilizing motion epochs identified on any one of all channels of data acquired as the tPCA is performed on all data channels simultaneously. Thus, the tPCA is applied to the motion artifacts at 5, 15 and 20 s that did not pass the objective criteria for motion artifacts for this specific channel but did pass for other channels.

**Fig. 3:**
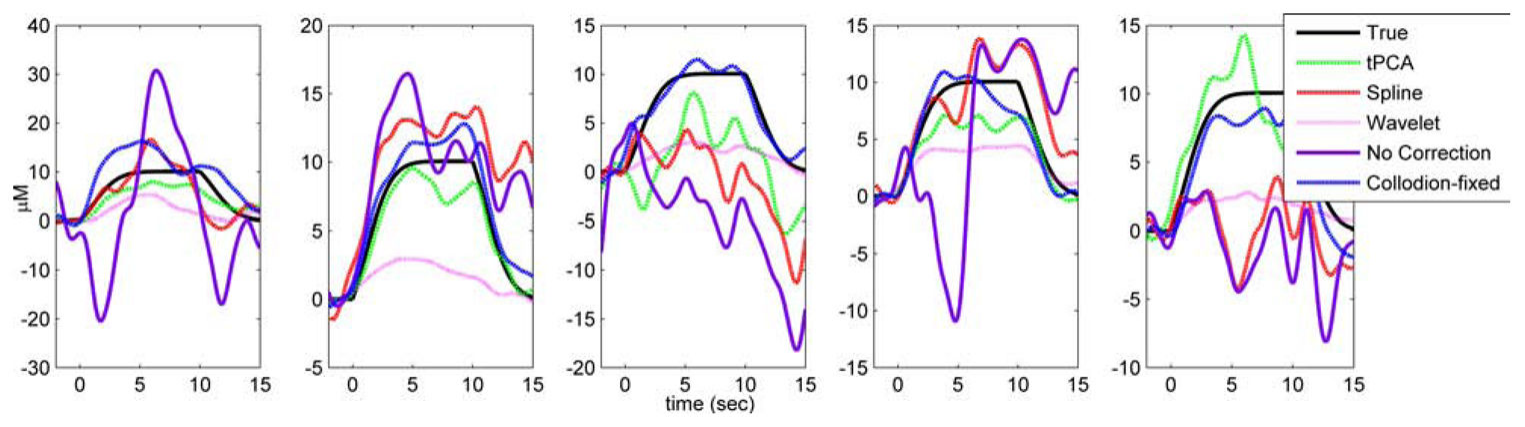
The HRFs (in micromolars) for one run from each subject obtained by block averaging the uncorrected data (velcro probe: purple line; collodion-fixed probe: blue-dashed line), tPCA corrected (green), spline interpolation corrected (red) and wavelet-based filter corrected data (pink). The true HRF is shown in black.

The HRFs obtained for one run from each subject after the application of tPCA, spline interpolation and wavelet-based filtering, are compared in Fig. 3 with the true HRF and the HRF obtained with no motion correction. tPCA and spline interpolation produce results closer to the true HRF. While the wavelet-based filtering captures the shape of the HRF well, it is often lower in amplitude. Consistent with the results obtained by Cooper *et al.*,^20^ application of the motion artifact correction algorithms produces better results than that obtained with no motion correction.

**Fig. 4:**
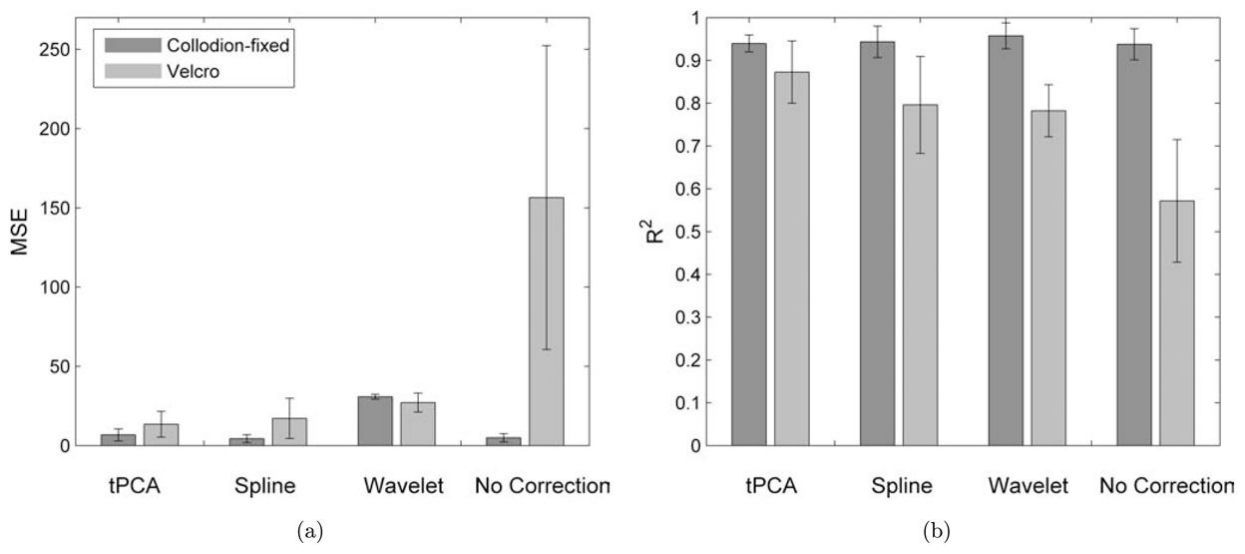
The group mean and standard deviation for MSE (a) and Pearson’s correlation coefficient (b) obtained from the channels for the Collodion-fixed fiber probe (green) and the Velcro-based probe (blue) corrected for motion artifacts by tPCA, spline interpolation, wavelet-based filtering and compared against no correction.

The MSE and *R*^2^ obtained for each motion artifact correction method are shown for the Velcro and collodion-fixed fiber probe in Fig. 4. The tPCA method produced the largest improvement in both MSE and *R*^2^ for the Velcro probe. The MSE obtained for tPCA was statistically lower than wavelet-based correction and no correction (paired *t*-test, *p*-value < 0.01) and was marginally smaller than the MSE for spline interpolation (paired *t*-test, *p*-value < 0.08). tPCA also showed the highest correlation with the true HRF. *R*^2^ for tPCA was statistically higher than the rest of the motion correction methods and no motion correction (paired *t*-test, *p*-value < 0.01). As the collodion-fixed fiber probe minimizes motion artifact in the first place, it produces a remarkable improvement in signal quality even without the application of any motion correction method. The MSE was statistically lower and the *R*^2^ was statistically higher for collodion-fixed fiber probe with no motion correction when compared to that of the Velcro probe treated with any of the motion correction algorithms (paired *t*-test, *p*-value < 0.01).

## 4. Discussion

In this study, we introduced targeted PCA as a new approach for motion artifact correction of NIRS signals. Our results show that the new approach produced statistically better results in HRF recovery, in terms of the MSE and Pearson’s correlation coefficient, compared with spline and wavelet-based correction methods. The advantage of tPCA over regular PCA is that it targets only the epochs of data where a motion artifact is present. As the hemodynamic response can simultaneously appear across multiple channels, a regular PCA could accidentally remove the desired signal from the data and thus result in an underestimation of the true HRF. This undesirable result is less likely when only motion artifact epochs are targeted. However, in this study, synthetic HRFs were added at random times to a real *in vivo* NIRS signal containing motion artifacts. For this reason, the motion artifact segments and the HRF were not temporally correlated. It is likely that the tPCA method would not perform as well in a situation where cerebral activation and motion artifacts are correlated, for instance for a study requiring subjects to speak aloud^21^ or move their body in response to a stimulus.

As exemplified in Fig. 2, the tPCA overcomes some limitations that are apparent with the spline and wavelet motion correction algorithms. It is evident that the wavelet method does an excellent job of removing high-frequency motion artifacts. It is also evident that low-frequency artifacts introduced by the motion artifacts are not removed. As a result, motion artifacts that produce a baseline shift in the optical density will remain after wavelet motion correction and thus have the potential to negatively impact the estimation of the evoked hemodynamic response to brain activation. There is clearly an opportunity to modify the wavelet correction algorithm to also remove these baseline shifts. Spline and tPCA explicitly correct for baseline shifts before and after a motion artifact epoch. The spline method, however, does not reduce the high-frequency motion artifacts as much as tPCA. Although wavelet filtering performs relatively well at the Velcro side, it significantly increases the MSE for the collodion-fixed probe at the suggested wavelet threshold. This is due to the fact that wavelet filtering also removes the physiology from the data, hence reduces the HRF amplitude drastically. Increasing this number could be an option, however, it has the disadvantage of missing some of the motion artifacts.

One interesting result is that the HRF recovered with the collodion-fixed fiber probe, which is much more resilient to motion artifacts, without the application of any motion artifact correction algorithm is as good as or even better than the HRF recovered from motion artifact corrected signal from the Velcro-based probe. This reinforces the point that better HRF estimation will be obtained by utilizing a probe that is more resilient to motion artifacts than by relying on motion correction algorithms. Thus, if the experimental study permits and motion artifacts are expected, the use of a collodion-fixed fiber probe may be desirable. If the use of a collodion-fixed probe is not feasible, in cases where the motion artifacts and the stimulus onsets are not correlated, the tPCA can help recover a HRF that is almost as good as that obtained with the collodion-fixed probe.

## References

[1] Jöbsis FF (1977). Noninvasive, infrared monitoring of cerebral and myocardial oxygen sufficiency and circulatory parameters. Science.

[2] Aldrich CJ, Wyatt JS, Spencer JAD, Reynolds EOR, Delpy DT (1994). The effect of maternal oxygen administration on human fetal cerebral oxygenation measured during labour by near infrared spectroscopy. Obstet Gynecol.

[3] Nollert G, Jonas RA, Reichart B (2000). Optimizing cerebral oxygenation during cardiac surgery: A review of experimental and clinical investigations with near infrared spectrophotometry. Thorac Cardiovasc Surg.

[4] Villringer A, Chance B (1997). Non-invasive optical spectroscopy and imaging of human brain function. Trends Neurosci.

[5] Chance B (1989). Time resolved spectroscopic (TRS) and continuous wave spectroscopic (CWS) studies of photon migration in human arms and limbs. Adv Exp Med Biol.

[6] Sato H, Takeuchi T, Sakai KL (1999). Temporal cortex activation during speech recognition: An optical topography study. Cognition.

[7] Steinhoff BJ, Herrendorf G, Kurth C (1996). Ictal near infrared spectroscopy in temporal lobe epilepsy: A pilot study. Seizure.

[8] Watanabe E, Maki A, Kawaguchi F, Yamashita Y, Koizumi H, Mayanagi Y (2000). Noninvasive cerebral blood volume measurement during seizures using multichannel near infrared spectroscopic topography. J Biomed Opt.

[9] Eschweiler GW, Wegerer C, Schlotter W, Spandl C, Stevens A, Bartels M, Buchkremer G (2000). Left prefrontal activation predicts therapeutic effects of repetitive transcranial magnetic stimulation (rTMS) in major depression. Psychiatry Res.

[10] Fallgatter AJ, Roesler M, Sitzmann L, Heidrich A, Mueller TJ, Strik WK (1997). Loss of functional hemispheric asymmetry in Alzheimer’s dementia assessed with near-infrared spectroscopy. Brain Res Cogn Brain Res.

[11] Khan B, Wildey C, Francis R, Tian F, Delgado MR, Liu H, Macfarlane D, Alexandrakis G (2012). Improving optical contact for functional near infrared brain spectroscopy and imaging with brush optodes. Biomed Opt Express.

[12] Coyle SM, Ward TE, Markham CM (2007). Brain-computer interface using a simplified functional near-infrared spectroscopy system. J Neural Eng.

[13] Strangman G, Boas DA, Sutton JP (2002). Non-invasive neuroimaging using near-infrared light. Biol Psychiatry.

[14] Yücel MA, Selb J, Boas DA, Cash SS, Cooper RJ (2013). Reducing motion artifacts for long-term clinical NIRS monitoring using collodion-fixed, prism-based optical fibers. Neuroimage.

[15] Zhang Q, Brown EN, Strangman GE (2007). Adaptive filtering for global interference cancellation and real-time recovery of evoked brain activity: A Monte Carlo simulation study. J Biomed Opt.

[16] Molavi B, Dumont GA (2012). Wavelet-based motion artifact removal for functional near-infrared spectroscopy. Physiol Meas.

[17] Zhang Y, Brooks DH, Franceschini MA, Boas DA (2005). Eigenvector-based spatial filtering for reduction of physiological interference in diffuse optical imaging. J Biomed Opt.

[18] Scholkmann F, Spichtig S, Muehlemann T, Wolf M (2010). How to detect and reduce movement artifacts in near-infrared imaging using moving standard deviation and spline interpolation. Physiol Meas.

[19] Izzetoglu M, Chitrapu P, Bunce S, Onaral B (2010). Motion artifact cancellation in NIR spectroscopy using discrete Kalman filtering. Biomed Eng Online.

[20] Cooper RJ, Selb J, Gagnon L, Phillip D, Schytz HW, Iversen HK, Ashina M, Boas DA (2012). A systematic comparison of motion artifact correction techniques for functional near-infrared spectroscopy. Front Neurosci.

[21] Brigadoi S, Ceccherini L, Cutini S, Scarpa F, Scatturin P, Selb J, Gagnon L, Boas DA, Cooper RJ (2013). Motion artifacts in functional near-infrared spectroscopy: A comparison of motion correction techniques applied to real cognitive data. Neuroimage.

[22] Brainard DH (1997). The psychophysics toolbox. Spatial Vision.

[23] Huppert TJ, Diamond SG, Franceschini MA, Boas DA (2009). HomER: A review of time-series analysis methods for near-infrared spectroscopy of the brain. Appl Opt.

[24] van der Zee P, Cope M, Arridge SR, Essenpreis M, Potter LA, Edwards AD, Wyatt JS, McCormick DC, Roth SC, Reynolds EO (1992). Experimentally measured optical pathlengths for the adult head, calf and forearm and the head of the newborn infant as a function of inter optode spacing. Adv Exp Med Biol.

